# Chemistry and Biochemistry of Dietary Polyphenols

**DOI:** 10.3390/nu2121231

**Published:** 2010-12-10

**Authors:** Rong Tsao

**Affiliations:** Guelph Food Research Centre, Agriculture & Agri-Food Canada, 93 Stone Road West, Guelph Ontario, N1G 5C9, Canada; Email: rong.cao@agr.gc.ca; Tel.: +1-519-780-8062; Fax: +1-519-829-2600

**Keywords:** polyphenols, phenolics, phenolic acids, flavonoids, extraction, separation, antioxidant, cell signal modulation

## Abstract

Polyphenols are the biggest group of phytochemicals, and many of them have been found in plant-based foods. Polyphenol-rich diets have been linked to many health benefits. This paper is intended to review the chemistry and biochemistry of polyphenols as related to classification, extraction, separation and analytical methods, their occurrence and biosynthesis in plants, and the biological activities and implications in human health. The discussions are focused on important and most recent advances in the above aspects, and challenges are identified for future research.

## 1. Introduction

Dietary polyphenols have received tremendous attention among nutritionists, food scientists and consumers due to their roles in human health. Research in recent years strongly supports a role for polyphenols in the prevention of degenerative diseases, particularly cancers, cardiovascular diseases and neurodegenerative diseases [1,2]. Polyphenols are strong antioxidants that complement and add to the functions of antioxidant vitamins and enzymes as a defense against oxidative stress caused by excess reactive oxygen species (ROS). Although most of the evidence of the antioxidant activity of polyphenols is based on *in vitro* studies, increasing evidence indicates they may act in ways beyond the antioxidant functions *in vivo*. Modulation of cell signaling pathways by polyphenols may help significantly to explain the mechanisms of the actions of polyphenol-rich diets [3,4]. In the meantime, chemically, polyphenols are a group of natural compounds with phenolic structural features. It is a collective term for several sub-groups of phenolic compounds, however, use of the term “polyphenols” has been somewhat confusing and its implied chemical structures are often vague even to researchers. Studies have also shown that different polyphenol subgroups may differ significantly in stability, bioavailability and physiological functions related to human health. This review intends to clarify some confusion related to the classification of polyphenols, to identify problems and examine methods in the characterization of polyphenols. Discussions about other aspects of polyphenol chemistry and biochemistry, *i.e.*, biosynthesis in plants, and possible biological roles and implications to human health, can help establish new directions for future research.

## 2. Classification of Polyphenols

Dietary phenolics or polyphenols constitute one of the most numerous and widely distributed groups of natural products in the plant kingdom. More than 8000 phenolic structures are currently known, and among them over 4000 flavonoids have been identified [5,6,7]. Although polyphenols are chemically characterized as compounds with phenolic structural features, this group of natural products is highly diverse and contains several sub-groups of phenolic compounds. Fruits, vegetables, whole grains and other types of foods and beverages such as tea, chocolate and wine are rich sources of polyphenols. The diversity and wide distribution of polyphenols in plants have led to different ways of categorizing these naturally occurring compounds. Polyphenols have been classified by their source of origin, biological function, and chemical structure. Also, the majority of polyphenols in plants exist as glycosides with different sugar units and acylated sugars at different positions of the polyphenol skeletons. To simplify the discussions, classification of polyphenols in this review will be done according to the chemical structures of the aglycones. 

### 2.1. Phenolic Acids

**Figure 1:**
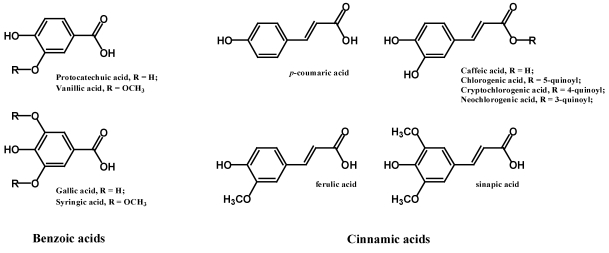
Typical phenolic acids in food: Left, Benzoic acids; right, Cinnamic acids.

Phenolic acids are non-flavonoid polyphenolic compounds which can be further divided into two main types, benzoic acid and cinnamic acid derivatives based on C1–C6 and C3–C6 backbones (Figure 1). While fruits and vegetables contain many free phenolic acids, in grains and seeds—particularly in the bran or hull—phenolic acids are often in the bound form [8,9,10]. These phenolic acids can only be freed or hydrolyzed upon acid or alkaline hydrolysis, or by enzymes. 

### 2.2. Flavonoids

**Figure 2:**
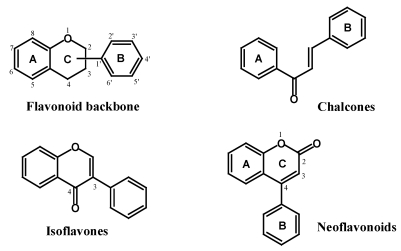
Basic flavonoid structures.

Flavonoids have the C6–C3–C6 general structural backbone in which the two C6 units (Ring A and Ring B) are of phenolic nature (Figure 2). Due to the hydroxylation pattern and variations in the chromane ring (Ring C), flavonoids can be further divided into different sub-groups such as anthocyanins, flavan-3-ols, flavones, flavanones and flavonols. While the vast majority of the flavonoids have their Ring B attached to the C2 position of Ring C, some flavonoids such as isoflavones and neoflavonoids, whose Ring B is connected at the C3 and C4 position of Ring C, respectively, are also found in plants. Chalcones, though lacking the heterocyclic Ring C, are still categorized as members of the flavonoid family. These basic structures of flavonoids are aglycones; however, in plants, most of these compounds exist as glycosides. Biological activities of these compounds, including antioxidant activity, depend on both the structural difference and the glycosylation patterns.

#### 2.2.1. Isoflavones, Neoflavonoids and Chalcones

**Figure 3:**
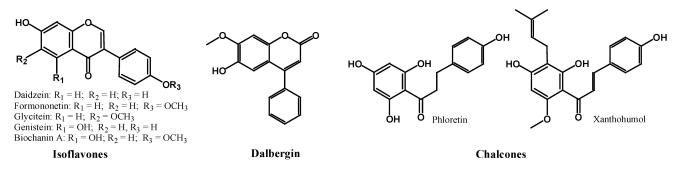
Typical isoflavones, neoflavones and chalcones found in food.

Isoflavones have their Ring B attached to the C3 position of Ring C (Figure 3). They are mostly found in the leguminous family of plants. Since beans, particularly soybean, are a major part of the diet in many cultures, the role of isoflavones have, thus, great impact on human health. Genistein and daidzein are the two main isoflavones found in soy along with glycetein, biochanin A and formononetin [11,12] (Figure 3). They are also found in red clovers [13]. All these isoflavone aglycones are mostly found as 7-*O*-glucosides and 6"-*O*-malonyl-7-*O*-glucosides. Neoflavonoids are not often found in food plants, but dalbergin is the most common and relatively widely distributed neoflavone in the plant kingdom [14]. The open-ring chalcones are found in fruits such as apples [15] and hops or beers [16].

#### 2.2.2. Flavones, Flavonols, Flavanones and Flavanonols

**Figure 4:**
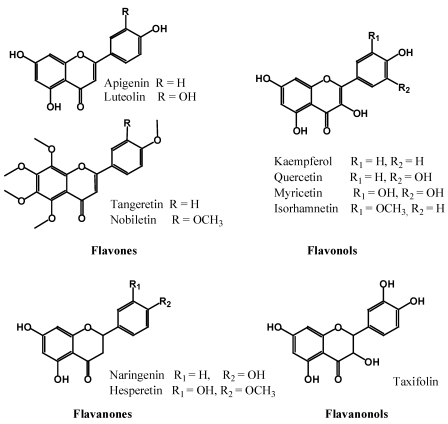
Flavones, flavonols, flavanones and flavanonols.

These flavonoid subgroups are the most common, and almost ubiquitous, throughout the plant kingdom (Figure 4). Flavones and their 3-hydroxy derivatives flavonols, including their glycosides, methoxides and other acylated products on all three rings, make this the largest subgroup among all polyphenols. The most common flavonol aglycones, quercetin and kaempferol, alone have at least 279 and 347 different glycosidic combinations, respectively [17,18,19]. The number of flavanones, and their 3-hydroxy derivatives (flavanonols, which are also referred to as dihydroflavonols) identified in the last 15 years has significantly increased. Some flavanones have unique substitution patterns, e.g., prenylated flavanones, furanoflavanones, pyranoflavanones, benzylated flavanones, giving a large number of substituted derivatives of this subgroup. A well known flavanonol is taxifolin from citrus fruits [20,21]. 

#### 2.2.3. Flavanols and Proanthocyanidins

**Figure 5:**
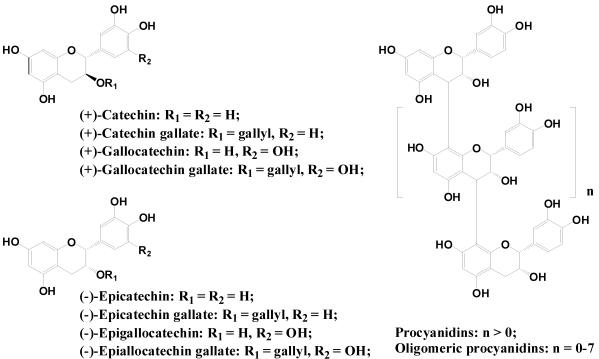
Flavanols and procyanidins.

Flavanols or flavan-3-ols are often commonly called catechins. Different from most flavonoids, there is no double bond between C2 and C3, and no C4 carbonyl in Ring C of flavanols. This and the hydroxylation at C3 allows flavanols to have two chiral centers on the molecule (on C2 and C3), thus four possible diastereoisomers. Catechin is the isomer with *trans* configuration and epicatechin is the one with *cis* configuration. Each of these two configurations has two steroisomers, *i.e.*, (+)-catechin, (−)-catechin, (+)-epicatechin and (−)-epicatechin. (+)-Catechin and (−)-epicatechin are the two isomers often found in food plants (Figure 5). Flavanols are found in many fruits, particularly in the skins of grapes, apple and blueberries [15]. Monomeric flavanols (catechin and epicatechin), their derivatives (e.g., gallocatechins) are the major flavonoids in tea leaves and cacao bean (chocolate) [22,23]. Catechin and epicatechin can form polymers, which are often referred to as proanthocyanidins because an acid-catalyzed cleavage of the polymeric chains produces anthocyanidins (Figure 5). 

**Figure 6:**
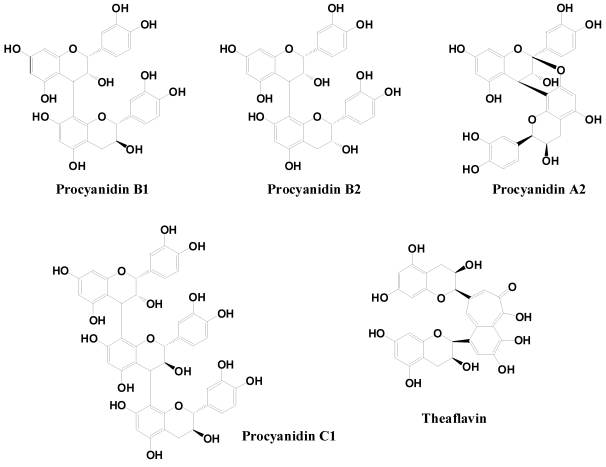
Typical procyanidin dimers, trimers and theaflavin.

Proanthocyanidins are traditionally considered to be condensed tannins. Flavanols and oligomers (containing 2–7 monomeric units) are known as strong antioxidants, which have been associated with several potential health benefits. Depending on the interflavanic linkages, oligomeric proanthocyanidins can be A-type structure in which monomers are linked through C2–*O*–C7 or C2–*O*–C5 bonding, or B–type in which C4–C6 or C4–C8 are common. Procyanidin C1 is a trimer. Also, tea flavanols can form unique dimers like theaflavin when fermented (Figure 6). 

#### 2.2.4. Anthocyanidins

**Figure 7:**
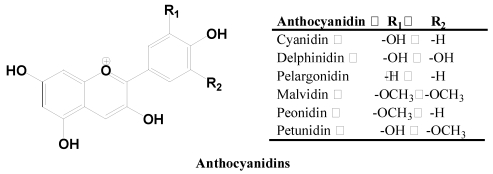
Major anthocyanidins.

Anthocyanidins are the principal components of the red, blue and purple pigments of the majority of flower petals, fruits and vegetables, and certain special varieties of grains, e.g., black rice (Figure 7). Anthocyanidins in plants mainly exist in glycosidic forms which are commonly referred to as anthocyanins. Cyanidin, delphinidin and pelargonidin are the most widely found anthocyanidins, along with more than two dozen other monomeric anthocyanidins (a total of 31 anthocyanidins) [24]. In fact, 90% of anthocyanins are based on cyanidin, delphinidin and pelargonidin and their methylated derivatives [24]. A total of more than 500 anthocyanins are known depending on the hydroxylation, methoxylation patterns on the B ring, and glycosylation with different sugar units [17,25]. The color of anthocyanins is pH-dependent, *i.e.*, red in acidic and blue in basic conditions. However, other factors such as degree of hydroxylation, or methylation pattern of the aromatic rings, and the glycosylation pattern, *i.e.*, sugar *vs.* acylated sugar can also affect the color of anthocyanin compounds. Anthocyanins are chemically stable in acidic solutions.

### 2.3. Polyphenolic Amides

**Figure 8:**
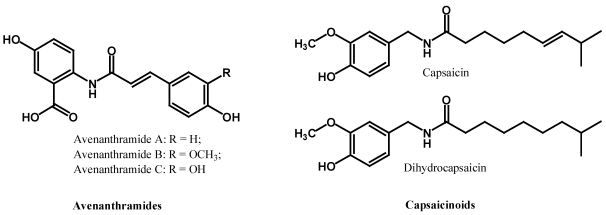
Polyphenol amides.

Some polyphenols may have N-containing functional substituents. Two such groups of polyphenolic amides are of significance for being the major components of common foods: capsaicinoids in chili peppers [26] and avenanthramides in oats [27] (Figure 8). Capsaicinoids such as capsaicin are responsible for the hotness of the chili peppers but have also been found to have strong antioxidant and anti-inflammatory properties, and they modulate the oxidative defense system in cells. Antioxidant activities including inhibition of LDL oxidation by avenanthramides have also been reported.

### 2.4. Other Polyphenols

**Figure 9:**
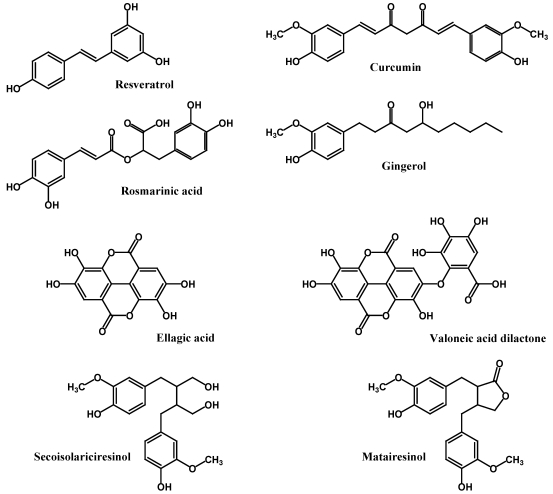
Other important polyphenols.

In addition to the phenolic acids, flavonoids and phenolic amides, there are several non-flavonoid polyphenols found in foods that are considered important to human health. Among these, resveratrol is unique to the grapes and red wine; ellagic acid and its derivatives are found in berry fruits, e.g., strawberries and raspberries, and in the skins of different tree nuts. Lignans exist in the bound forms in flax, sesame and many grains; structures shown below (Figure 9) are hydrolysis products. Curcumin is a strong antioxidant from turmeric (Figure 9). Rosmarinic acid is a dimer of caffeic acid, and ellagic acid is a dimer of gallic acid. While both gallic acid and ellagic acid are found in the free forms, their glucose esters, a group known as hydrolysable tannins, also exist in different plants. These compounds may possess anti-nutritive properties, thus will not be discussed in detail in this review.

## 3. Biosynthesis of Polyphenols

Biosynthesis of polyphenols has been thoroughly discussed by many experts in the field and readers are referred to recent reviews and books for details [17,28]. Like all phenolic compounds, phenolic acids such as gallic acid and cinnamic acid are considered to be metabolites of the shikimate pathway. Biosynthesis of complex polyphenols such as flavonoids is linked to primary metabolism through plastid and mitochondrial derived intermediates, each requiring export to the cytoplasm where they are incorporated into separate parts of the molecule. The aromatic ring B and the chromane ring are considered to originate from the amino acid phenylalanine, itself a product of the shikimate pathway, whereas Ring A from three units of malonyl-CoA [17,29]. These three malonyl-CoA units are added through sequential decarboxylation condensation reactions, which initiates flavonoid biosynthesis. 

Phenylalanine ammonia lyase (PAL) is a key enzyme of the phenylpropanoid pathway which catalyzes the conversion of phenylalanine to cinnamate, which then leads to the C6–C3 structures. The final intermediate 4-coumaroyl-CoA and three molecules of malonyl-CoA are then condensed to yield the first flavonoid structure naringenin chalcone by the enzyme chalcone synthase (CHS). Chalcone is isomerized by chalcone flavanone isomerase (CHI) to a flavanone. This flavanone intermediate is pivotal because it is essentially from where all classes of flavonoids—including their subgroups—branch out. Chalcone is also where isoflavones and coumestrols branch out from through different enzymes including CHI and isoflavone synthase (IFS). For example, the intermediate (2S)-flavanones are catalyzed by flavanone 3-hydroxylase (F3H) to dihydroflavonols, which are then reduced by dihydroflavonol reductase (DFR) to flavan-3,4-diols (leucoanthocyanins), which are converted to anthocyanidins by anthocyanidin synthase (ANS). Glucosylation of flavonoids is catalyzed by glucosyltransferase [17,30]. Understanding the biosynthetic pathways of polyphenols can help the breeding program for designer foods with enhanced polyphenol content and health benefits [31].

## 4. Separation, Identification and Analysis of Polyphenols

As discussed above, even though polyphenols share the common phenolic feature, due to the structural diversity, these phytochemicals vary significantly in their physicochemical properties. Owing to the chemical complexity and the frequent occurrence of polyphenols in plants, extraction, separation, identification and analysis of polyphenols remain as challenging as ever, despite the recent advances in new instrumentation. The challenge is multiplied when the complex glycosylation and polymerization patterns and the various food matrices are considered. While it is nearly impossible to develop a protocol for all polyphenols, there are some general approaches to these important aspects of polyphenol research. Many good reviews and books are available on this subject, so only a brief summary will be covered here [32,33,34,35].

**Figure 10:**
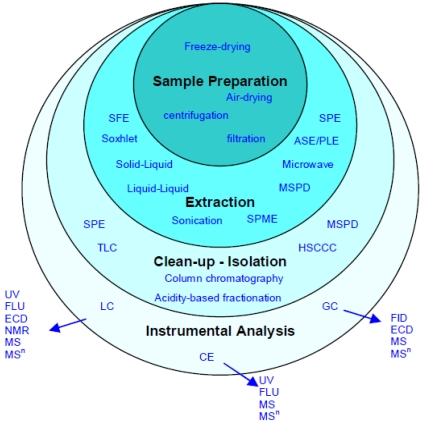
Schematic of strategies for the determination of phenolic acids and flavonoids in biological fluids, beverages, plants, and food. Abbreviations: SFE, supercritical fluid extraction; MSPD, matrix solid-phase dispersion; SPME, solid-phase microextraction; ASE/PLE, accelerated solvent extractin/pressurized liquid extraction; HSCCC, high-speed counter-current chromatography; TLC, thin layer chromatography; FL, fluorescence;FID, flame ionization detection; ECD, electron capture detection (GC)/electrochemical detector (LC); CE, capillary electrophoresis (modified from [33]).

Before polyphenols are extracted, samples containing these compounds must be collected, reserved and prepared properly. It is generally understood that samples (e.g., plants, foods, biological fluids) collected must represent the actual pool. Care must be taken to minimize the loss of compounds of interest during transportation and preservation of the samples. To avoid degradation of native polyphenols, samples are often dried, frozen or lyophilized before extraction because high moisture or water content aids enzyme activities [33] (Figure 10). Heating and exposure to light and oxygen may affect the polyphenolic composition in many cases; therefore high-temperature drying should be avoided as much as possible. Antioxidants such as butylated hydroxytoluene (BHT) and ascorbic acid are often added to samples to avoid oxidation of the polyphenols. Sample pre-treatment may be done by filtration and centrifugation as well [33] (Figure 10).

Many different extraction methods are available for different types of samples [32,33,34]. For the majority of plant originated food samples, solvent extractions such as liquid/liquid partitioning and solid/liquid extraction are most frequently employed in the laboratory. The phenolic nature of polyphenols makes them relatively hydrophilic, thus free polyphenols, including aglycones, glycosides, and oligomers, are extracted using water, polar organic solvents such as methanol, ethanol, acetonitrile and acetone, or their mixture of water. The liquid extracts are sometimes partitioned with solvents such as ethyl acetate, depending on the solubility of the target polyphenols. Also important is the pH of the extraction solvent. For polyphenols, most extractions are carried out under acidic conditions because they are generally more stable in low pH, and the acidic condition helps polyphenols to stay neutral, thus readily extracted into organic solvents. This is done using weak acid or low concentrations of a strong acid. High acid concentration can cause hydrolysis of glycosides or acylglycosides and thus may give different pictures of native polyphenol profiles. On the other hand, not all polyphenols exist in the free form. Phenolic acids such as ferulic acid and lignans in grains are often bound to structural materials. Hydrolysis using acid or alkaline releases these phenolics which are partitioned into ethyl acetate or n-butanol [8,9,10]. Sometimes, intentional hydrolysis is carried out to obtain aglycones with enzymes such as β-glucosidase, or with strong acid such as 2–4 M HCl at elevated temperature such as refluxing. This is necessary, particularly when the glycosylation patterns are extremely complex, and when standard reference materials of polyphenol glycosides are unavailable. The hydrolysis can simplify the chromatographic profile during separation, and aid quantification and structural identification of the polyphenols. 

**Figure 11:**
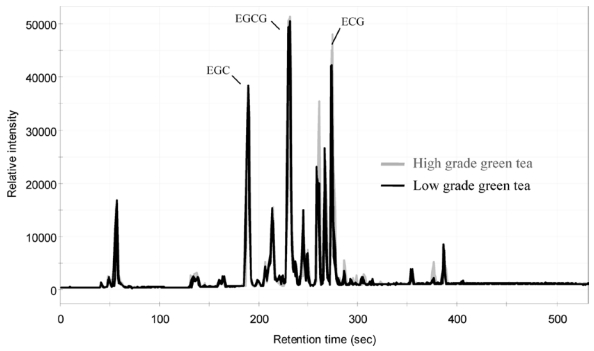
Total ion chromatograms of Japanese green tea dried leaves (high- and low–grade green teas). Key compounds for quality determination of green tea: (−)-epigallocatechin (EGC), (−)-epigallocatechin gallate (EGCG), and (−)-epicatechin gallate (ECG). Adapted from [38].

Spectrophotometric methods have been developed, and are still used for the estimation of total phenolic, total flavonoid and total anthocyanins contents [36,37]. While these methods are rapid and simple, they lack the specificity for individual compounds. Interferences from non-polyphenolic components of the sample can also cause false readings and thus lead to erroneous results. In order to accurately quantify and identify the individual polyphenols, these compounds must be separated first. Methods for separation using various chromatographic techniques have been reviewed, and their advantages and disadvantages discussed [32,33,34]. Reversed-phase high performance liquid chromatography (HPLC) coupled with a diode array detector (DAD) and/or mass spectrometric detector (LC-MS) is the most widely use analytical tool for quantification of polyphenols, although occasionally polyphenols such as isoflavones are derivatized to methyl esters and analyzed by gas chromatography (GC), or normal phase column for the separation of procyanidins [23] (Figure 10). In–depth review on methods related to separation and structural identification techniques is not the purpose of this paper, however, one recent development in chromatography and how it can be applied in polyphenol analysis is worth mentioning. The development of ultra high performance liquid chromatography (U-HPLC or UPLC) has significantly enhanced the performance of separation. Polyphenols can be separated with significantly enhanced efficiency and drastically reduced analytical time (to <1/10 of the time of a conventional HPLC) [38,39] (Figure 11).

To obtain the identity of a polyphenol, the most common method is to compare the retention time of a particular compound with the standard. The DAD can collect UV/visible spectral data as the compounds are separated, thus when a peak matches the retention time and the UV/Vis spectrum of a standard, it can be tentatively identified [40]. On-line LC-MS, particularly techniques such as tandem mass spectrometry that employs collision-induced dissociation (CID), can provide sufficient information for the final confirmation of most known polyphenols found in foods [41]. Glycosides of the same aglycone, such as quercetin glycosides, have similar UV/Vis spectrum, but can differ slightly in retention time, thus identified. When standards are not available, to identify different glycosides, peaks can be separated, hydrolyzed, and analyzed for sugar composition [15]. Identifications using these techniques are usually positive, however, care must be taken when studying polyphenols of different plant samples. 

## 5. Biological Roles and Implications (Mainly on Antioxidant Activities)

### 5.1. Antioxidant Activity

Polyphenols are secondary metabolites that plants produce to protect themselves from other organisms. Dietary polyphenols have been shown to play important roles in human health. High intake of fruits, vegetables and whole grains, which are rich in polyphenols, has been linked to lowered risks of many chronic diseases including cancer, cardiovascular disease, chronic inflammation and many degenerative diseases [1,2]. Recent studies have revealed that many of these diseases are related to oxidative stress from reactive oxygen and nitrogen species. Phytochemicals, especially polyphenols, are the predominant contributor to the total antioxidant activities of fruits, rather than vitamin C [42]. Polyphenols have been found to be strong antioxidants that can neutralize free radicals by donating an electron or hydrogen atom. The highly conjugated system and certain hydroxylation patterns such as the 3-hydroxy group in flavonols are considered important in the antioxidant activities. Polyphenols suppress the generation of free radicals, thus reducing the rate of oxidation by inhibiting the formation of or deactivating the active species and precursors of free radicals. More frequently, they act as direct radical scavengers of the lipid peroxidation chain reactions (chain breakers). Chain-breakers donate an electron to the free radical, neutralizing the radicals and themselves becoming stable (less reactive) radicals, thus stopping the chain reactions [43,44,45].

In addition to radical scavenging, polyphenols are also known as metal chelators. Chelation of transition metals such as Fe^2+^ can directly reduce the rate of Fenton reaction, thus preventing oxidation caused by highly reactive hydroxyl radicals [44,46]. Polyphenols do not act alone. It has been found that polyphenols can actually function as a co-antioxidant, and are involved in the regeneration of essential vitamins [47]. 

Several *in vitro* antioxidant model systems have been developed to evaluate the total antioxidant activities. Although these methods are limited in terms of similarity to the mechanisms of antioxidant actions in a biological system, collectively, they may portray well how polyphenols function as antioxidants, and thus shed light on the actual role of polyphenols in human health. Pros and cons of the *in vitro* chemical models have been thoroughly discussed [48], and are beyond the scope of this review. Caution also must be taken when antioxidant activities are evaluated by *in vitro* models. From the chemistry point of view, molecules of polyphenols, once they have donated an electron or hydrogen atom, themselves become free radicals, given enough concentration, they therefore can potentially cause pro-oxidant activities. However, whether or not such pro-oxidant activity will occur *in vivo* and cause harm to human is a question, and further research is needed [49].

### 5.2. Beyond the Usual Antioxidant Activity

In addition to the above possible mode of antioxidant actions, other mechanisms such as inhibition of xanthine oxidase and elevation of endogenous antioxidants are also considered important [50]. Polyphenols can induce antioxidant enzymes such as glutathione peroxidase, catalase and superoxide dismutase that decompose hydroperoxides, hydrogen peroxide and superoxide anions, respectively, and inhibit the expression of enzymes such as xanthine oxidase [51].

While direct and indirect antioxidant activities of polyphenols may play important roles in reducing oxidative stress via the above mentioned mechanisms, the actual roles at the cellular level of these compounds may be more complicated. There is an emerging view that phytochemicals, particularly polyphenols and their *in vivo* metabolites, do not act as conventional hydrogen- or electron-donating antioxidants but may exert modulatory actions in cells through actions at protein kinase and lipid kinase signaling pathways [3]. Although polyphenols such as flavonoids can be absorbed through the gastrointestinal tract, the concentrations in plasma are low, usually less than 1 μmol/L, in part because of rapid metabolism by human tissues [49,52,53,54]. This is too low a concentration for most polyphenols to exhibit any significant and direct antioxidant activities, thus some researchers have even taken the view that it is unlikely that polyphenols act as antioxidants *in vivo*. Thus, attention should be brought to functions beyond the usual antioxidant activities [49,3]. Nutrigenomics, as a result of such views, has emerged as a new multidisciplinary area of research, not only on polyphenols, but on phytochemicals as a whole. Effects on biomarkers involved in the above and other pathways can lead to changes in cellular functions, thus potential health benefits. Williams *et al.* [3] also suggest that “a clear understanding of the mechanisms of action of flavonoids, either as antioxidants or modulators of cell signaling, and the influence of their metabolism on these properties are key to the evaluation of these potent biomolecules as anticancer agents, cardioprotectants, and inhibitors of neurodegeneration” [3]. Future research on polyphenols is no doubt headed in the same direction.
